# Influence of Compounding Parameters on the Tensile Properties and Fibre Dispersion of Injection-Moulded Polylactic Acid and Thermomechanical Pulp Fibre Biocomposites

**DOI:** 10.3390/polym14204432

**Published:** 2022-10-20

**Authors:** Chiara Zarna, Sandra Rodríguez-Fabià, Andreas T. Echtermeyer, Gary Chinga-Carrasco

**Affiliations:** 1 Department of Mechanical and Industrial Engineering, NTNU, Richard Birkelandsvei 2B, 7491 Trondheim, Norway; 2 RISE PFI AS, Høgskoleringen 6b, 7491 Trondheim, Norway

**Keywords:** biocomposite, wood fibres, compounding, material characterisation

## Abstract

Thermomechanical pulp (TMP) fibres can serve as renewable, cost-efficient and lightweight reinforcement for thermoplastic polymers such as poly(lactic acid) (PLA). The reinforcing ability of TMP fibres can be reduced due to various factors, e.g., insufficient dispersion of the fibres in the matrix material, fibre shortening under processing and poor surface interaction between fibres and matrix. A two-level factorial design was created and PLA together with TMP fibres and an industrial and recyclable side stream were processed in a twin-screw microcompounder accordingly. From the obtained biocomposites, dogbone specimens were injection-moulded. These specimens were tensile tested, and the compounding parameters statistically evaluated. Additionally, the analysis included the melt flow index (MFI), a dynamic mechanical analysis (DMA), scanning electron microscopy (SEM) and three-dimensional X-ray micro tomography (X-μCT). The assessment provided insight into the microstructure that could affect the mechanical performance of the biocomposites. The temperature turned out to be the major influence factor on tensile strength and elongation, while no significant difference was quantified for the tensile modulus. A temperature of 180 °C, screw speed of 50 rpm and compounding time of 1 min turned out to be the optimal settings.

## 1. Introduction

Biocomposites from wood-fibre-reinforced thermoplastic polymer have shown potential to be a replacement for fossil-based short-fibre-reinforced composites, such as glass-fibre-reinforced polyolefins. PLA is a biobased and biodegradable thermoplastic polymer and thermomechanical pulp (TMP) fibres have proven their ability to reinforce PLA [1]. Additionally, recycled wood particles obtained from sawmill side streams can be used as a cost-efficient filler material, as a stiffness enhancement and as a potential MFI enhancement [2] that could be beneficial for biocomposites processed by injection moulding, and reused side streams can help to improve circularity and waste management [3,4]. The overall purpose is to create more sustainable, lightweight and cost-efficient products. Such biocomposites, mainly processed by injection moulding, compression moulding or extrusion can serve as nonstructural parts in the automotive sector, in building and construction, for furniture and household items as well as sports and leisure equipment [5]. However, the poor compatibility between hydrophilic fibres and the hydrophobic matrix may hinder the full potential of wood-fibre-reinforced biocomposites. Additionally, the relatively low degradation temperature of wood fibres (200 °C) limits the processibility of such biocomposites [6,7,8]. Natural raw materials generally show a greater variety in their properties than man-made materials. This might negatively affect the reproducibility and consistency of biocomposite parts.

For preparing biocomposites, one common procedure is to mix dry polymer and possibly a compatibiliser powder before adding wood fibres, e.g., TMP fibres. Additionally, it can be necessary to dry the raw materials prior to processing to eliminate moisture that evaporates during the heat treatment and might cause inclusions and defects. Furthermore, moisture can affect the dimensional stability of TMP fibres and disturb the fibre–matrix interface [9]. In addition, some polymers (e.g., PLA) degrade in the presence of water [10,11].

The dried and mixed biocomposite components can either be fed directly into a melt extruder, or processed into pellets beforehand by melt compounding, pressing and chopping. Through melting and mechanical shearing, the mixture is then compounded and homogenised. The compounding process can be repeated several times, but fibre damage and polymer degradation must be considered. Drying TMP fibres makes them brittle and fragile. This causes fibre damage and shortening during compounding [7,10]. Wet TMP fibres are not that susceptible to damage development, but they agglomerate and cannot be dispersed properly. As shown by Yang et al. [12], to some extent, fibre agglomeration may affect the flexural properties of the biocomposite more negatively than fibre shortening. This was true for compounding wet pulp and high-density polyethylene. However, when oven drying and pelleting of the fibres was done prior to compounding, a reduction in fibre length resulted in an improvement of fibre dispersion and thus a lower flexural strength of the biocomposite [12]. Wood fibres generally have shown to change their mechanical properties and morphology during melt processing. The property changes were mainly related to temperature and exposure time [9,13]. High temperature and long residence times were also found to have a negative impact on the tensile strength of neat polypropylene due to thermal degradation and hydrolysis due to the presence of moisture [14]. On the other hand, to some extent, a higher screw speed could have a positive effect on the tensile strength [15] and fibre dispersion [16,17].

It can be differentiated between continuous and discontinuous melt compounding. In continuous compounding there is a continuous material flow along different mixing elements, e.g., kneading blocks, conveying elements or tooth mixing elements that are connected in series to accomplish certain objectives. The duration of the treatment is dependent on the feeding rate [18,19]. In discontinuous compounding the biocomposite components remain in the barrel and are mixed by two conveying screws for a certain duration of time after being released [20]. Discontinuous microcompounding is especially beneficial if small batches are required for early material development. However, using different processing equipment might lead to different biocomposite properties even when the same parameter settings are used. This applies for upscaling from a discontinuous batch compounder to a continuous compounder for larger quantities or when switching to a similar machine from another manufacturer due to specific dimensions of the barrel and the screws [21].

When compounding TMP fibres and biopolymers, the aim is to achieve a homogeneous dispersion of fibres in the matrix while maintaining the fibre length. To take full advantage of TMP fibre reinforcements, it is of major importance to select suitable processing parameters [9]. The discontinuous compounding process is controlled by the compounding time, screw rotation speed, compounding temperature and the screw design [22]. Adjusting these parameters may affect the mechanical properties of the resulting biocomposites [19] and is a trade-off between sufficient dispersion and material degradation or damage. Increasing the temperature, screw speed and compounding time decreases the viscosity of the polymer, accelerates the process and increases the probability of homogeneous dispersion, respectively. However, elevated temperatures may cause a degradation of the biocomposite components. Elevated screw speed increases the shear and friction forces which can cause fibre damage and uncontrolled internal heating. A longer compounding time results in longer exposure duration of the biocomposite components to heat and mechanical forces. Thus, thermal, and mechanical damage can evolve more significantly. Therefore, studies to optimise the compounding process and gain information on how each compounding parameter influences the biocomposite properties are crucial [23]. The parameters might not only be optimised towards desired biocomposite properties but also towards other goals such as minimum energy consumption or time efficiency [24].

In this work, a design of experiments (DoE) approach is used to find optimal parameters for compounding TMP fibres, an industrial side stream (S) from fibre boards and PLA in a twin-screw microcompounder. However, it should be noted that the presented results are not only limited to the biocomposite formulation used in this study but can serve as a baseline for other formulations of lignocellulosic fibres and polymers having a similar processing temperature as PLA. The purpose of the study was to provide a method for performing a parameter optimisation for biocomposite processing using a microcompounder and highlighting the impact of exposure time, temperature and screw speed on the biocomposite properties. Importantly, we hypothesised that wax-containing side stream could be used to tailor the MFI. It was thus important to verify whether the compounding variables affected the performance of the side stream as an MFI modifier. Further, microcompounding is greatly used for research purposes [25,26,27,28] because of the possibility of producing small batch sizes below 50 g. However, to the best of our knowledge there is a lack of comprehensive studies addressing the effect of microcompounding parameters on the biocomposite properties. The interaction of temperature and residence time and the sensitivity of natural fibre biocomposites on these factors was investigated and reviewed [9,29]. The presented DoE approach aims to clarify whether and how exposure time, temperature and screw speed affect the biocomposite tensile properties and morphology.

## 2. Materials and Methods

### 2.1. Raw Materials

**Figure 1:**
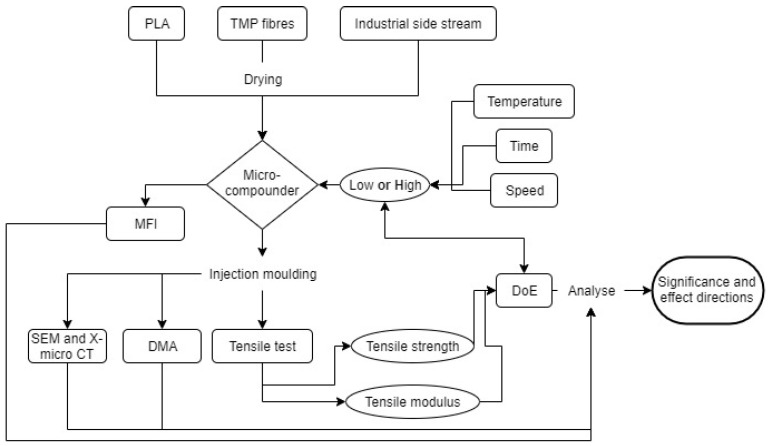
Flow chart to illustrate the research method.

The PLA used in this study was an Ingeo 4043D grade (NatureWorks, Minnetonka, MN, USA). The side stream was collected from the production plant at Alloc AS (Lyngdal, Norway). A chemical characterisation of this side stream can be found in [2]. The purpose of introducing this industrial side stream was to enhance the usage of waste material and further explore its beneficial effect on the MFI [2]. Spruce TMP fibres were prepared by Norske Skog Saugbrugs (Halden, Norway). The pulp was granulated to a size of <8 mm as described in [2]. The research method of the present study is illustrated using a flow chart (Figure 1).

### 2.2. Compounding

The side stream (S) was ground using a 30-mesh sieve in a Thomas Wiley Mini-Mill cutting mill (Thomas Scientific, Swedesboro, NJ, USA). The side stream powder and TMP fibres were dried for 1 h at 105 °C. PLA was dried for 4 h at 50 °C. The raw materials were compounded in an Xplore twin-screw microcompounder (Xplore Instruments BV, Sittard, The Netherlands). Eight series of biocomposites were compounded using different parameter settings by adjusting the compounding time, the screw rotation speed and the compounding temperature (Table 1). The chosen temperature range was restricted by the minimum processing temperature of PLA (180 °C) [30] recommended by the manufacturer and the degradation temperature of the TMP fibres (200 °C) [7]. The screw rotation speed and compounding time were chosen as low as possible to prevent harming the biocomposite raw materials by excessive mechanical shear forces and exposure to elevated temperatures. The goal was to maximise fibre dispersion while minimising thermal degradation and mechanical damages of the biocomposite components. The formulation of 70 wt.% PLA/20 wt.% TMP/10 wt.% S was equal for every batch. For each series three independent batches were prepared with the microcompounder and mixed randomly before further processing.

### 2.3. Injection Moulding

An Xplore injection moulding system (Xplore Instruments BV, Sittard, The Netherlands) was used to form tensile test specimens for mechanical tensile testing. Eight test specimens per series were prepared. The injection temperature was 190 °C and the mould temperature was 30 °C.

### 2.4. Tensile Tests

The injection-moulded dogbone specimens were mechanically tensile-tested with an MTS Criterion 42 503E testing machine (MTS, Eden Prairie, MN, USA) and a load cell of 5 kN, using an extensometer (MTS 632.29F-30) with 5 mm gauge length. The test speed was 2 mm/min. The modulus was calculated from the linear slope between two points on the stress–strain curves at a strain of 0.25% and 0.5% according to ISO 527-2:2012.

### 2.5. Statistical Analysis

The effect of compounding parameters on the tensile properties of injection-moulded specimens was assessed as part of a $2^{3}$ full factorial design, i.e., three factors (compounding parameters from Table 1) at two levels (low and high from Table 1) with five repetitions per series. As a response, the tensile strength and tensile modulus were assigned. The statistical analysis of the standardised effects and interactions was done using Minitab^®^ 19.2020.1 software. Prior to the analysis of variance (ANOVA) a normality test (Shapiro–Wilk) and a homoscedasticity test (Levene) was performed to ensure the data sets were following the normal distribution and the variances were equal. To compare each series to each other and assess the statistical difference between groups, a post hoc test (Tukey method) was used. It was determined which factors had a statistically significant influence on the tensile properties and which series were significantly different from each other. A significance level of 0.05 was chosen. Additionally, interaction plots were used to show how the relationship between one compounding parameter and the tensile properties depended on the value of another compounding parameter [31]. To find the *p*-value, the *F*-test from the analysis of variance was used:

(1) $$F=\frac{\mathrm{Effect}\mathrm{variance}}{\mathrm{Error}\mathrm{variance}}$$

The procedure for performing the *F*-test is explained in [32]. The *F*-ratio (Equation (1)) was obtained by dividing the mean squares between the factors by the mean squares within the groups (factors). The *p*-value could then be found in *F* tables for desired probabilities (here 5%) and degrees of freedom [32].

### 2.6. Melt Flow Index

The melt flow index (MFI) of samples from series 1/50/180 and 2/50/200 was measured with a Melt Flow Index-Deluxe (model no: MFI—DX, Presto Stantest Private Limited, Faridabad, India). These samples were chosen because they resulted in the highest and lowest tensile strength, respectively. A temperature of 190 °C was applied, the preheating of a sample was 5 min and a weight of 2.16 kg was used. Ten measurements were undertaken for each sample, and this was repeated two times on different batches. There is a significant difference in sample mass when the sample is taken right after the beginning or towards the end of the material extrusion. The results presented here were obtained from samples taken right after starting the test.

### 2.7. Scanning Electron Microscopy

The fracture surfaces of injection-moulded samples from series 1/50/180 and 2/50/200 were assessed with scanning electron microscopy (SEM). The fracture area was coated with a layer of gold and visualised in secondary electron mode. SEM was conducted with a Hitachi scanning electron microscope (SU3500, Hitachi High-Tech Corporation, Tokyo, Japan). The acceleration voltage and working distance were 5 kV and 5–10 mm, respectively.

### 2.8. Dynamic Mechanical Analysis

A dynamic mechanical analysis (DMA) was performed on injection-moulded dog-bone specimens of series 1/50/180 and 2/50/200. The tests were performed in accordance with ISO 6721-11:2019 (method A) on a Gabo Eplexor 1500 N test machine from NETZSCH (Selb, 95100, Germany). The dog-bone specimens were used in axial tension mode in a temperature range of 25 °C to 100 °C. The tests were performed at a constant frequency of 10 Hz, a static strain of 0.2%, a dynamic strain of 0.1% and a heat rate of 5 K/min.

### 2.9. X-ray Micro-Computed Tomography

The fibre dimensions were assessed on injection-moulded samples from series 1/50/180 and 2/50/200 with X-μCT [33]. The samples were imaged with a Xradia MicroXCT-400 tomograph (XRadia, Concord, CA, USA) with a 1.1 μm pixel size. The X-ray tube voltage was set to 30 kV and the power to 3 W. A total of 1881 projections were acquired with 8 s exposure time and 10× magnification. Three-dimensional volume images were constructed using an algorithm from [34]. A cropped region of the image was edited by bilateral and high-pass filtering [35]. Fibres, matrix and voids were segmented by thresholding according to [36]. To segment the agglomerates, individual fibres were erased with a morphological opening filter, and the pores in the agglomerates were closed by a morphological closing filter. The fibre lengths were determined using the constrained path transform [37] and the fibre orientation using the structure tensor method [38]. By applying the approach of Miettinen et al. [33], the fibre length and orientation were combined into fibre property distributions. With the local thickness algorithm [39], agglomerates were characterised by measuring the volume and the surface area.

## 3. Results and Discussion

### 3.1. Tensile Tests

**Figure 2:**
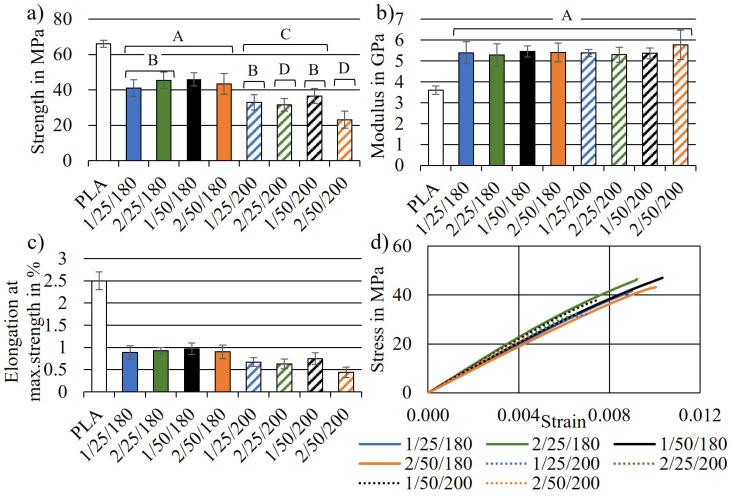
Results from tensile tests of the biocomposite series. (**a**) Tensile strength with indication of homogeneous groups A–D, (**b**) tensile modulus, (**c**) elongation at maximum stress and (**d**) representative stress–strain curve of each series.

The tensile test results of injection-moulded dog bones, prepared by using eight different compounding parameter combinations (Table 1) are presented in Figure 2. Additionally, groups of responses with similar characteristics could be identified using the Tukey method. Four groups named A, B, C and D were identified. The series means that do not share a group letter were significantly different.

The tensile modulus was similar for all eight biocomposite series, as seen in Figure 2b. No significant effect could be observed, as indicated by the grouping information. All series means were assigned to the same group A. Regarding the tensile strength, four data groups were identified. Series compounded at 180 °C (group A) generally showed a higher tensile strength than the series compounded at 200 °C (group C and D). The same trend was observed for the elongation at maximum strength. The highest tensile strength was found for series 1/50/180 with (45.96 ± 3.68) MPa and the lowest for series 2/50/200 with (23.16 ± 4.77) MPa. Additionally, series 2/50/200 (Group D) showed a significantly lower tensile strength than the other series compounded at 200 °C (Group C). The combined average tensile modulus of all series was found to be (5416 ± 143) MPa. Series 1/50/180 and 1/50/200 resulted in the highest tensile strength inside their data group. Both were compounded for 1 min at 50 rpm. Thus, for achieving the highest possible tensile strength, this setting seemed to be more appropriate than the others. The strength of all series was lower than the one of neat PLA. This was most probably related to the presence of waxes in S. The waxes might have disturbed the interfacial adhesion between PLA and the fibres and thus acting as a defect rather than a reinforcement [2]. Additionally, the addition of stiff fibres and particles to a ductile matrix such as PLA restricts the mobility and deformation of the matrix leading to a reduced strain to failure [40].

In biocomposites, the modulus is mainly influenced by the fibre and matrix modulus, the fibre volume fraction and orientation but only to a much lesser degree by the fibre length [41]. The tensile modulus of a TMP-fibre-reinforced biocomposite can be calculated as suggested by Thomason [42]:

(2) $$E=V_{m}E_{m}\eta_{1}+V_{f}E_{f}\eta_{0}$$

In Equation (2), $V_{i}$ and $E_{i}$ are the volume fraction and elastic modulus. *i* = *m*, *f* refers to the matrix or the fibres. $\eta_{1}$ is obtained by the shear lag theory developed by Cox and refers to the morphology of the fibres. $\eta_{0}$ is an orientation factor [42]. For injection-moulded short-fibre composites, the fibre orientation is mostly considered as being random due to the different orientations of the fibres in the midplane and close to the mould surface [43]. According to Equation (2), the modulus gets reduced with lower fibre length. The factor of fibre length is eliminated when using a model for particulate-reinforced composites [44]:

(3) $$E=\frac{V_{f}^{0.67}E_{m}}{1-V_{m}^{0.33}\left(1-\frac{E_{m}}{E_{f}}\right)}+\left(1-V_{f}^{0.67}\right)E_{m}$$

The tensile strength is more sensitive to the fibre length or length-to-width ratio. If the fibre length is below the critical load transfer length, the fibre might rather act as a defect than a reinforcement [10,45]. The tensile strength can be predicted by:

(4) $$\sigma =V_{f}\sigma_{f}x_{1}x_{2}+V_{m}\sigma_{m}$$

**Figure 3:**
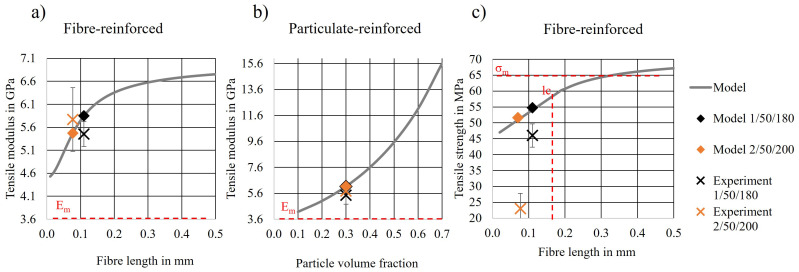
Micromechanical models for (**a**) Tensile modulus for short-fibre-reinforced composites (Cox) with varying fibre length, (**b**) tensile modulus for particulate-reinforced composites (Mital) with varying particle volume fraction and (**c**) tensile strength of short-fibre-reinforced composites (Bowyer and Bader) with varying fibre length.

For illustrating the effect of fibre length or particle volume fraction on the tensile modulus and strength with the proposed models, the parameters according to Table 2 were applied and plotted in Figure 3. The values in Table 2 refer to the biocomposite used in this study. The material properties of the matrix (PLA) were taken from the above-presented measurements and the TMP fibre properties were taken from the literature, as indicated in Table 2. The fibre morphology was assessed using X-μCT. The fibre orientation was considered random [43] and the corresponding factors were taken from the literature [10,42,45]. The experimentally obtained data points (cross markers) of the series resulting in the lowest (2/50/200) and the highest (1/50/180) tensile strength are plotted in each model graphic. Additionally, the tensile strength and stiffness values for the corresponding biocomposite predicted by the model are indicated by a rhombus marker. The grey graph is a plot of the Equations (2)–(4) over the fibre length or fibre volume fraction. All parameters were kept constant except for the fibre length *l* in $\eta_{1}$ (Figure 3a,c) or the fibre volume fraction $V_{f}$ (Figure 3b).

According to the micromechanical model in Equation (2) (Figure 3a), longer fibres are expected to stiffen the matrix to some extent. However, this was not true for the biocomposites presented here. As indicated by the rhombus marker, the modulus of both series was similar despite the difference in fibre length. Regarding the effect of the volume fraction (Figure 3b) of particulate reinforcements, it can be observed that the fibre or particle volume fraction had a much greater impact on the composite stiffness. Since in the biocomposites presented here, the volume fraction of the TMP fibres and S particles was the same in all series, it is understandable that the effect of marginally different fibre lengths was negligible. Both models predicted the modulus fairly well with an error of about 10%. However, the error was the least when applying the model for particulate reinforcements (Equation (3)) to series 2/50/200 and the one for fibre reinforcements (Equation (2)) to series 1/50/180, indicating that the TMP fibres in series 2/50/200 where probably broken down to a particle-like shape.

Figure 3c shows the modelled and experimentally obtained tensile strength of the biocomposite in relation to the TMP fibre length. Although the model (Equation (4)) overestimated the experimental results from both series 1/50/180 and 2/50/200, the measured and modelled tensile strengths of series 1/50/180 deviated by only 14% when applying the micromechanical model for fibre lengths below the critical load transfer length (*l* < $l_{c}$). However, in the case of 2/50/200, the analysis showed that not only the fibre length reduction caused the comparatively low tensile strength of biocomposites from this series, but porosity and insufficient fibre–matrix interaction probably also contributed to the major reduction of tensile strength. Importantly, the higher temperature in combination with a higher screw speed and compounding time might have led to the thermal degradation of matrix and fibres, fibre shortening due to elevated shear forces and probably the evaporation of moisture and volatilisation contributing to void formations and a reduced fibre–matrix interaction [1,13,47].

### 3.2. Statistical Analysis

The *p*-values obtained from the test for normality and homoscedasticity were greater than the confidence level of 0.05 for all tested sets of data. Thus, the data of the tensile strength and modulus followed the normal distribution for each series and the variances between the two levels of each factor were equal for both responses. The *p*-values found for the influence of the compounding parameters on the tensile properties with a confidence level of 0.05 are provided in Table 3.

**Figure 4:**
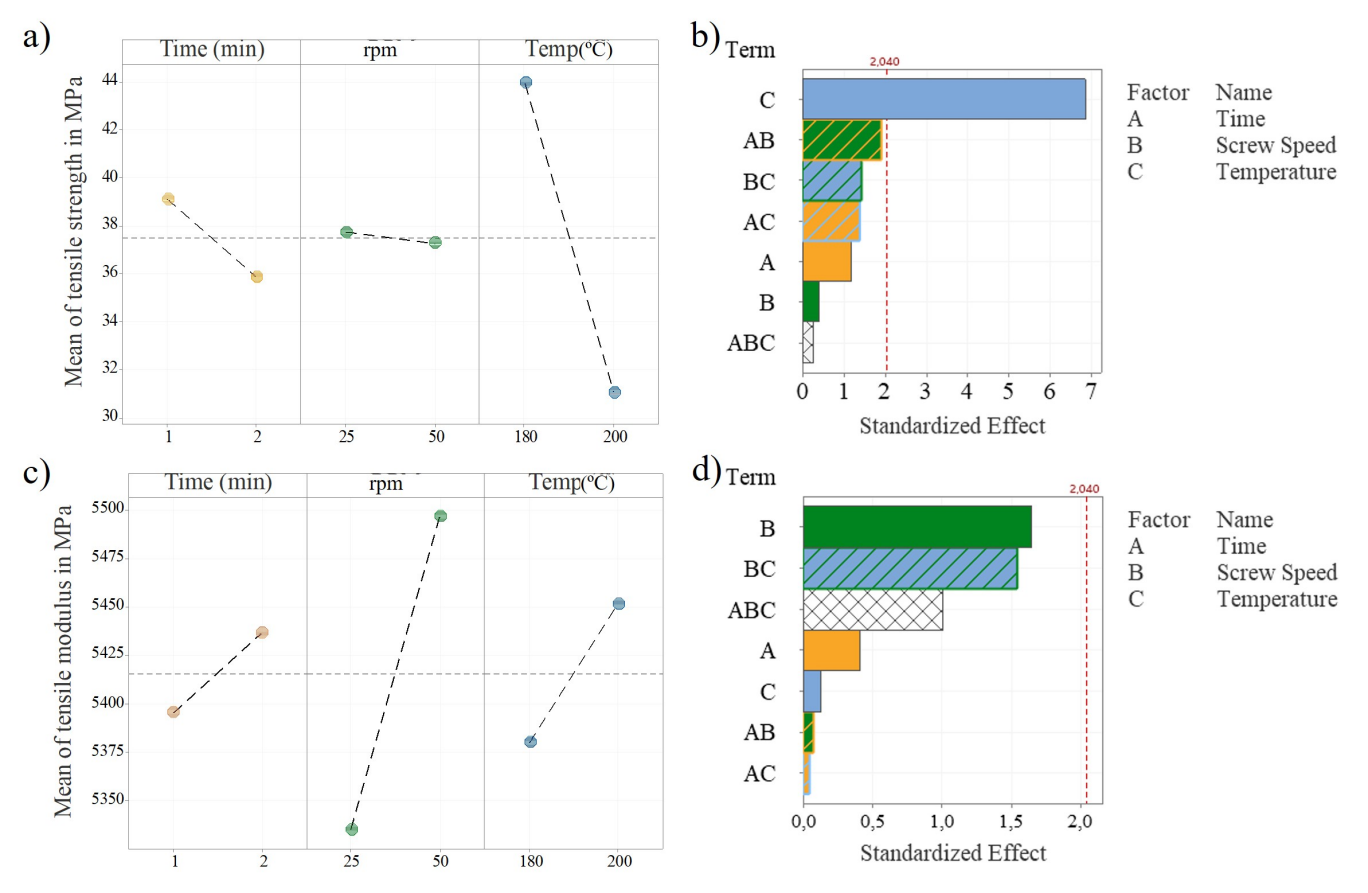
Main effect plots for the response of (**a**) tensile strength and (**c**) tensile modulus and Pareto charts of standardised effects for (**b**) tensile strength and (**d**) tensile modulus.

The only *p*-value less than 0.05 was found for the influence of temperature on the tensile strength. As already indicated by the bar graph in Figure 2b, no significant influence of any compounding parameter on the tensile modulus was found. The combined effects of two or all three parameters were not significant either. A Pareto chart of standardised effects and main effect plots for the tensile strength and modulus are presented in Figure 4.

**Figure 5:**
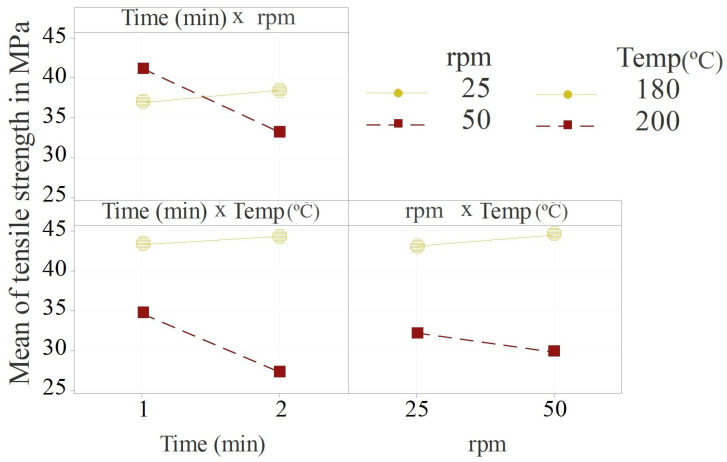
Interaction plots for the tensile strength.

Figure 4b and d show whether a factor had a significant effect on the response or not. If the red dashed line is crossed by a bar, the corresponding factors had a significant effect on the response. This was only valid for the effect of compounding temperature on the tensile strength. The combined factors showed a relatively similar effect magnitude on the tensile strength. The screw speed had the least effect, but it became more prominent in combination with time or temperature. Regarding the effect plot for the tensile strength (Figure 4a), it can be observed that the higher value or level of each factor had a negative effect on the tensile strength, meaning that an elevated compounding time, screw speed and temperature led to a lower tensile strength of the biocomposite. An opposite but not significant effect can be observed for the tensile modulus (Figure 4c). The screw speed had the largest effect on the tensile modulus followed by the combined effect of temperature and screw speed. The temperature and combination of screw speed and temperature and time and temperature had the least effect on the tensile modulus. The screw speed most probably contributed to the fibre dispersion and potential fibre damage or shortening. It was previously shown that the fibre or particle size had no significant effect on the tensile modulus [40]. However, a slight trend of decreasing modulus with increasing particle size was observed [40], consistent with the results presented here. Since no significance was found for the tensile modulus response, interaction plots are only shown and discussed for the tensile strength response (Figure 5).

The upper left plot in Figure 5 shows the combined effect direction of compounding time and screw speed on the tensile strength. The yellow data points correspond to the lower level of speed and the red data points to the higher level of speed. It can be concluded that when a lower screw speed (25 rpm) was applied, the effect of the compounding time was very little. When a higher screw speed (50 rpm) was applied, the effect of time became more prominent. The same trends can be observed for the combined effects of compounding time and temperature and screw speed and temperature. This shows that the effects intensified each other.

From the tensile test results and the statistical analysis, it can be concluded that a 180 °C compounding temperature, 50 rpm screw speed and 1 min compounding duration were the optimal parameter settings for achieving the highest tensile strength of the tested biocomposite. Similar compounding parameters were suggested by Lu et al. [48] for a wood fibre high-density polyethylene composite. The results of the present study can serve as a baseline for compounding similar lignocellulosic-reinforced biocomposites. Instead of 50 rpm and 1 min (series 1/50/180), 25 rpm and 2 min (series 2/25/180) resulted in a very similar average tensile strength (Figure 2). However, this was not valid when applying a higher compounding temperature of 200 °C. Here, the maximum tensile strength could only be obtained by applying 50 rpm and 1 min (series 1/50/200). Not-dried biocomposite components may (inter)act differently. Wet lignocellulosic fibres might be less brittle and sensitive to shear forces, but the fibre–matrix surface interaction may be disturbed by the presence of water [12]. Varying the processing procedure [12] or applying these compounding parameters to other compounding equipment, especially to large-scale continuous extruders might lead to different mechanical properties than the ones presented here. This is due to potentially different screw shapes and dimensions leading to different mechanical shear forces, internal friction, etc. [21]. However, it can be assumed that the effect directions of the compounding parameters also remain similar when different equipment is used.

### 3.3. Melt Flow Index

Based on the results obtained from the tensile tests and the statistical analysis, an MFI analysis is presented for the series with the highest (1/50/180) and the lowest tensile strength (2/50/200). The biocomposite from series 1/50/180 was measured to have an MFI of (4.73 ± 1.79) g/10 min. Series 2/50/200 was not measurable because the biocomposite was not able to exit the nozzle of the MFI measurement equipment even when higher forces (5 kg) were applied.The MFI tester nozzle was clogged, and volatilisation was visible. The other three series compounded at 200 °C (1/25/200, 2/25/200, 1/50/200) were also not measurable, and a similar volatilisation was observed. Neat PLA has an MFI of 6 g/10 min, according to its technical data sheet [30]. Incorporating TMP fibres into a PLA matrix typically reduces the MFI [4]. However, the side stream in the biocomposite formulation presented here contained waxes [2] that may have contributed to reducing the interfacial adhesion between the PLA and the side stream particles. The side stream may, on the one hand, contribute to an increased MFI but on the other hand, to a reduction of tensile strength [2,49]. An increased MFI is beneficial with respect to the further processing of the biocomposite, e.g., injection moulding [2]. Typically, the MFI rises when harsher heat treatment or multiple cycles of heat treatment are applied due to chain scission [50]. It was therefore unexpected that the MFI of series 2/50/200 was lower than the MFI of series 1/50/180. The waxes might have been partially volatilised during the harsher heat treatment of series 2/50/200 thus eliminating the beneficial contribution of the waxes for increasing the MFI.

### 3.4. Dynamic Mechanical Analysis

**Figure 6:**
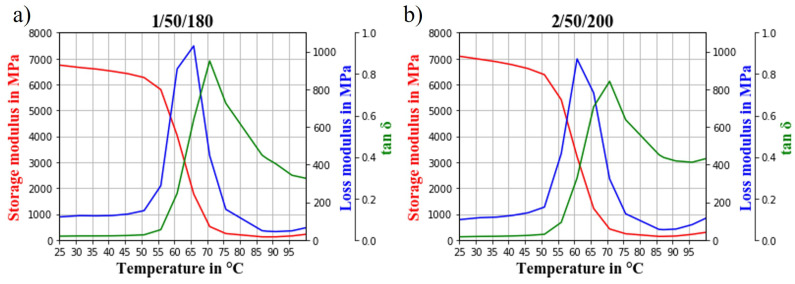
Storage modulus, loss modulus and tanδ obtained from DMA temperature sweep test of (**a**) series 1/50/180 and (**b**) series 2/50/200.

Injection-moulded dog-bone samples of series 1/50/180 and 2/50/200 were subjected to a DMA temperature sweep test to investigate their viscoelastic behaviour and glass transition temperature (Tg). The resulting storage modulus $E^{\prime }$, loss modulus $E^{\prime \prime }$ and tan(δ) are presented in Figure 6.

The storage modulus corresponds to the ability of a material to store energy elastically, while the loss modulus is the viscous response to stress and is related to the energy dissipated per cycle of sinusoidal deformation. The ratio of loss and storage modulus is tan(δ), also called loss factor.

The loss factor tan(δ) of series 1/50/180 was higher than the tan(δ) of series 2/50/200, meaning that series 1/50/180 behaved more viscous and series 2/50/200 more elastically. The TMP fibres generally lead to a reduction of chain mobility, thus more energy is required for the transition from the glassy to the rubbery state [51]. This indicated a reduced chain mobility of series 2/50/200 compared to series 1/50/180. Fillers with a lower surface area per volume or insufficient fibre–matrix surface interaction are known for allowing a greater mobility of the biocomposite, leading to increased heat dissipation under deformation through applied stress to the biocomposite. This results in a higher loss modulus, as seen for series 1/50/180 [52,53,54]. The greater chain mobility observed for series 1/50/180 was already indicated by its comparatively high MFI, thus confirming the presented results.

Further, an increased storage modulus can be an indication of a uniform dispersion of fillers in a matrix material. This is related to the assumption that agglomerates or nonuniformly dispersed fibres would not restrict the movement of polymer chains as much as uniformly dispersed ones [12,52]. The storage modulus of series 2/50/200 was higher than the one of series 1/50/180, indicating more uniformly dispersed fibres in samples of series 2/50/200. This is expected since series 2/50/200 was compounded for a longer time and at higher temperature than series 1/50/180. The restriction in chain mobility due to uniform fibre dispersion and a potentially improved fibre–matrix surface interaction due to the volatilisation of waxes most probably explains the lower MFI of series 2/50/200 compared to 1/50/180 [55].

According to ISO 6721-11:2019, the glass transition temperature can be read from the peak of the loss modulus curve, at the inflection point of storage modulus or at the peak of the tan(δ) curve. A reduction in molecular weight due to polymer degradation under processing might lead to a lower Tg. Generally, the processing of PLA might increase crystallinity leading to a reduced mobility and an increased Tg [56]. When read from the peak of $E^{\prime \prime }$ and the inflection point of $E^{\prime }$, series 1/50/180 had a higher Tg (66 °C or 63 °C) than series 2/50/200 (61 °C or 60 °C). The peak of tan(δ) was at 71 °C for both series.

### 3.5. Scanning Electron Microscopy

**Figure 7:**
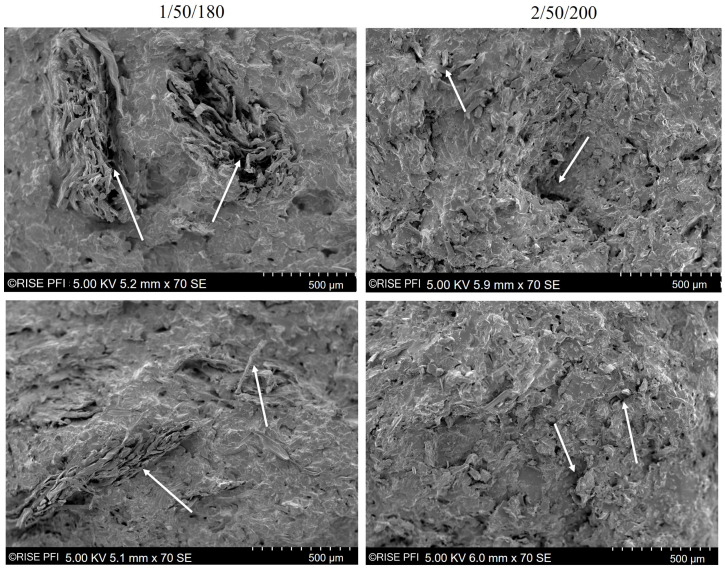
SEM images with 70× magnification of fractured surfaces of a tensile test specimens from series 1/50/180 (**left**) and series 2/50/200 (**right**).

Fractured tensile test specimens from the series 1/50/180 and 2/50/200 were investigated more closely. The fracture surfaces of the samples from series 1/50/180 (left) and one from series 2/50/200 (right) are shown in Figure 7.

In Figure 7 left, some fibre agglomerates are visible in the form of areas where fibres are accumulating without being surrounded by matrix material. Series 1/50/180 showed the highest tensile strength and elongation at maximum stress. However, the dispersion of fibres was not fully achieved as already indicated by the lower storage modulus of 1/50/180 compared to 2/50/200.

In Figure 7 right, the fracture surface of series 2/50/200 appears to be rougher and rugged compared to series 1/50/180. Fibre fragments and pores in the matrix are visible and it can clearly be seen that both fibres and matrix were damaged under compounding. The agglomerations visible on the fracture surface of 1/50/180 probably initiated the failure of the specimen. However, the matrix surrounding the agglomerates seems smoother and not as porous as the fracture surfaces of 2/50/200. This observation highlights the trade-off between homogeneous fibre dispersion and material degradation when compounding wood fibre biocomposites. As 1/50/180 was stronger and tougher than 2/50/200, it might be more beneficial to maintain the raw materials’ morphology to a certain extent at the expense of some fibre agglomerations.

### 3.6. X-ray Micro-Computed Tomography

**Figure 8:**
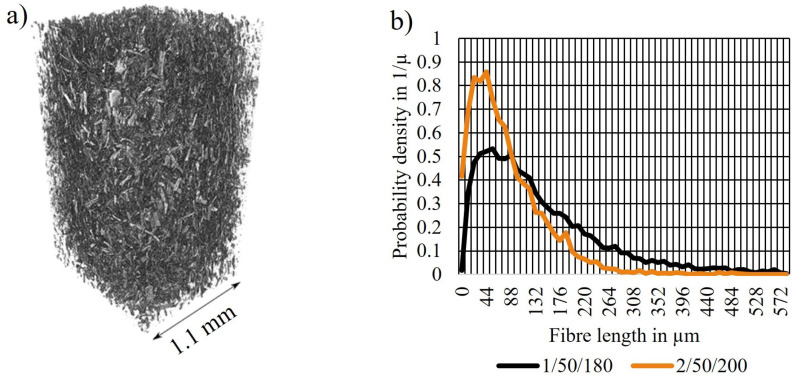
Results from X-μCT. (**a**) Visualisation of series 1/50/180, (**b**) fibre length distribution of series 1/50/180 and 2/50/200.

The fibre length distribution of series 1/50/180 and 2/50/200 was investigated on injection-moulded specimens using X-μCT (Figure 8).

As seen in Figure 8b, series 1/50/180 showed a higher probability for the presence of longer fibres than series 2/50/200. This agrees with the micromechanical modelling and tensile test results presented in Figure 3c. Since longer fibres were present in the biocomposites of series 1/50/180, more fibres could potentially transfer the load applied to the biocomposite. Although the TMP fibres were not fully dispersed in series 1/50/180 (as indicated by SEM images Figure 7 and DMA Figure 6), the applied compounding parameters were considered to be optimal. Based on the presented results from tensile tests and statistical analysis, it is not expected that either increasing compounding time, temperature or screw speed would contribute to a significantly higher tensile strength of the biocomposite. It was shown that a longer or stronger mixing may potentially improve the fibre dispersion but may damage the biocomposite components at the same time, resulting in a nonsignificant improvement of tensile strength. Potentially, other feeding techniques or mixing and pelletising prior to compounding could contribute to a more uniform dispersion [12,57].

## 4. Conclusions

An analysis of variance revealed that the compounding parameters had no influence on the tensile modulus. In terms of tensile strength, only the compounding temperature had a significant influence. Compounding the biocomposite at 200 °C led to a significantly lower tensile strength than compounding at 180 °C. Applying the high levels of all three factors yielded the biocomposite with the lowest tensile strength with a significant difference from all other data groups. The combined effects of parameters were not significant. However, when applying a screw speed of 50 rpm the influence of time became more prominent. A similar trend was observed for both applied temperatures.

The MFI revealed a severe difference in flow properties of series 1/50/180 (highest tensile strength) and 2/50/200 (lowest tensile strength). Series 2/50/200 was not measurable while series 1/50/180 had a similar MFI as neat PLA, most probably due to the wax-containing industrial side stream. In DMA, series 1/50/180 showed to be more viscous than series 2/50/200. Inhibited chain mobility in series 2/50/200 was an indicator for a more uniform filler dispersion. This was confirmed by SEM images that showed a rugged and porous fracture surface of biocomposites from series 2/50/200. Both, fibres and matrix were damaged, resulting in the low tensile strength. Biocomposites from series 1/50/180 showed a smoother surface but some fibre agglomerations were found. An X-μCT analysis confirmed the conclusions drawn from the previous analysis. The fibre lengths in the biocomposites from series 2/50/200 were generally shorter than those from series 1/50/180.

Finally, a micromechanical analysis was applied to explain, discuss and support the outcome of this study based on existing modelling approaches. The strong impact of degradation and porosity due to a higher compounding temperature and longer exposure time on the tensile strength was highlighted. We demonstrated that optimising the compounding process for biocomposites is crucial. The mechanical properties, microstructure and appearance of the biocomposite are highly dependent on the compounding parameters, especially the compounding temperature. For lignocellulosic-reinforced biocomposites, a temperature of 180 °C, screw speed of 50 RMP and a compounding time of 1 min can be suggested.

## Figures and Tables

**Table 1:** Compounding parameters to prepare biocomposites from 70 wt.% PLA/20 wt.% TMP/10 wt.% S.

Series Designation	Time (min)		Speed (rpm)		Temperature (°C)	
	Low	High	Low	High	Low	High
1/25/180	1		25		180	
2/25/180		2	25		180	
1/50/180	1			50	180	
2/50/180		2		50	180	
1/25/200	1		25			200
2/25/200		2	25			200
1/50/200	1			50		200
2/50/200		2		50		200

**Table 2:** Parameters used for the analytical models.

Parameter	Value	Unit	Clarification
$V_{m}$	0.7	-	Matrix volume fraction
$V_{f}$	0.3	-	Fibre volume fraction
$E_{m}$	3.6	GPa	Matrix tensile modulus
$E_{f}$	2	GPa	Fibre tensile modulus [46]
$\eta_{0}$	0.375	-	Fibre orientation factor [42]
$\eta_{1}$	$1-\frac{tanh(\beta l/2)}{\beta l/2}$	-	Fibre length factor [42]
β	$\frac{2}{d}{\left[\frac{2G_{m}}{E_{f}ln\left(\sqrt{\pi /X_{i}V_{f}}\right)}\right]}^{1/2}$	-	- [42]
$X_{i}$	4.0	-	Squared packing value [42]
$G_{m}$	$\frac{E_{m}}{2(1+\nu )}$	MPa	Matrix shear modulus
ν	0.3	-	Poisson’s ratio of matrix
$\sigma_{m}$	66	MPa	Matrix’s ultimate tensile strength
$\sigma_{f}$	500	MPa	Fibre’s ultimate tensile strength [10]
$\tau_{m}$	$\frac{\sqrt{2}}{3}\sigma_{m}$	MPa	Matrix shear strength
*d*	0.02	mm	Fibre diameter (measured with X-μCT)
$l_{c}$	$\frac{\sigma_{f}}{2\tau_{m}}d$	mm	Critical load transfer length [45]
$x_{1}$	0.167	-	Fibre orientation factor [10]
$x_{2}$	$\frac{l}{2l_{c}};l<l_{c}$, $1-\frac{l_{c}}{2l};l\geq lc$	-	Length factor [10]

**Table 3:** *p*-values for the influence of the compounding parameters on the tensile properties with a significance level of 0.05.

Factor	*p*-Value	
	Tensile Strength Response	Tensile Modulus Response
Temperature	≪ 0.05	0.683
Speed	0.695	0.110
Time	0.250	0.898
Time × Speed	0.065	0.940
Time × Temperature	0.175	0.962
Speed × Temperature	0.162	0.133
Time × Speed × Temperature	0.785	0.321

## Data Availability

Not applicable.

## Abbreviations

The following abbreviations are used in this manuscript:

ANOVA	Analysis of variance
DMA	Dynamic mechanical analysis
DoE	Design of experiments
PLA	Poly(lactic acid)
S	Industrial side stream
SEM	Scanning electron microscopy
Tg	Glass transition temperature
TMP	Thermomechanical pulp
X-μCT	X-ray microtomography
